# Repurposing Serotonin-Norepinephrine-Dopamine Reuptake Inhibitors for the Treatment of Stimulant Use Disorder

**DOI:** 10.1192/bjo.2026.11943

**Published:** 2026-06-30

**Authors:** Cassidy Keen, Andre Samir Ramkaran, Neran Mahadeosingh, Michelle A. Sahai

**Affiliations:** 1 Brunel University of London, Uxbridge, United Kingdom; 2 Royal College of Surgeons in Ireland, Dublin, Ireland

## Abstract

**Aims:**

Stimulant use disorder is associated with increasing morbidity, mortality, and high relapse rates, yet there is currently no approved pharmacotherapy. Relapse vulnerability is increasingly recognised as arising from dysregulation across dopaminergic, serotonergic, and noradrenergic neurocircuitry. Serotonin-Norepinephrine-Dopamine Reuptake Inhibitors (SNDRIs) modulate these monoaminergic pathways and represent a mechanism-informed pharmacological strategy. This review aimed to evaluate the neurobiological rationale, translational potential, and emerging clinical feasibility of repurposing SNDRIs for stimulant use disorder.

**Methods:**

A review was conducted integrating preclinical neuropharmacological evidence, human neuroimaging and behavioural studies, and clinical trial data examining monoamine transporter modulation. Evidence relating to dopamine transporter (DAT), serotonin transporter (SERT), and norepinephrine transporter (NET) function was analysed to evaluate reinforcement neurobiology, relapse vulnerability, pharmacokinetic characteristics, and clinical safety data from candidate SNDRIs including dasotraline, centanafadine, and ansofaxine.

**Results:**

Psychostimulant reinforcement is strongly associated with DAT occupancy and dopamine elevation within mesolimbic reward circuitry. However, relapse vulnerability is increasingly linked to serotonergic and noradrenergic dysregulation contributing to stress sensitivity, affective instability, and cue-driven drug seeking. Preclinical evidence demonstrates that NET inhibition reduces stress-induced and cue-mediated stimulant seeking, while SERT modulation influences impulsivity and negative affective states associated with relapse. SNDRIs produce sustained, moderate monoaminergic modulation that normalises hypodopaminergic states observed during stimulant withdrawal without producing rapid dopamine elevations associated with abuse liability. Pharmacokinetic data demonstrates extended elimination half-lives supporting once-daily dosing and improved treatment adherence. Clinical trials investigating SNDRIs in attention-deficit/hyperactivity disorder and major depressive disorder demonstrate favourable tolerability and sustained monoaminergic modulation in human populations. Advances in transporter structural modelling and computational pharmacology support the use of dynamic transporter-ligand profiling to stratify therapeutic development and improve prediction of abuse liability.

**Conclusion:**

SNDRIs demonstrate mechanistic plausibility and clinical feasibility as candidate pharmacotherapies for stimulant use disorder. Multi-transporter modulation may address both reward-driven reinforcement and relapse-related neuropsychiatric processes. However, current evidence remains indirect and derived primarily from mechanistic, preclinical, and non-addiction clinical populations. Targeted, stratified clinical trials in stimulant use disorder populations are required before treatment recommendations can be made.

